# Ferroptosis-related signaling pathways in cancer drug resistance

**DOI:** 10.20517/cdr.2024.151

**Published:** 2025-01-06

**Authors:** Yang Yang, Simin Yu, Wanyao Liu, Yi Zhuo, Chunrun Qu, Yu Zeng

**Affiliations:** ^1^Department of Neurosurgery, Xiangya Hospital, Central South University, Changsha 410008, Hunan, China.; ^2^XiangYa School of Medicine, Central South University, Changsha 410013, Hunan, China.; ^3^Department of Urology, Innovation Institute for Integration of Medicine and Engineering, West China Hospital, Sichuan University, Chengdu 610041, Sichuan, China.; ^4^First Clinical Department of Changsha Medical University, Changsha 410219, Hunan, China.; ^5^National Clinical Research Center for Geriatric Disorders, Xiangya Hospital, Central South University, Changsha 410008, Hunan, China.

**Keywords:** Ferroptosis, drug resistance, cancer

## Abstract

Ferroptosis is an iron-dependent form of programmed cell death induced by lipid peroxidation. This process is regulated by signaling pathways associated with redox balance, iron metabolism, and lipid metabolism. Cancer cells’ increased iron demand makes them especially susceptible to ferroptosis, significantly influencing cancer development, therapeutic response, and metastasis. Recent findings indicate that cancer cells can evade ferroptosis by downregulating key signaling pathways related to this process, contributing to drug resistance. This underscores the possibility of modulating ferroptosis as an approach to counteract drug resistance and enhance therapeutic efficacy. This review outlines the signaling pathways involved in ferroptosis and their interactions with cancer-related signaling pathways. We also highlight the current understanding of ferroptosis in cancer drug resistance, offering insights into how targeting ferroptosis can provide novel therapeutic approaches for drug-resistant cancers. Finally, we explore the potential of ferroptosis-inducing compounds and examine the challenges and opportunities for drug development in this evolving field.

## INTRODUCTION

Ferroptosis, a regulated cell death form first identified in 2012, is characterized by iron-driven lipid peroxidation^[1]^. Unlike apoptosis, necrosis, and autophagy, ferroptosis is distinguished by the accumulation of reactive oxygen species (ROS) and specific lipid peroxides, regulated primarily through key metabolic signaling pathways^[2]^. Central players in ferroptosis include but are not limited to the cystine/glutamate antiporter system (Sx C-), glutathione peroxidase 4 (GPX4), and nuclear factor E2-related factor 2 (NRF2 or NFE2L2), which collectively maintain redox homeostasis and protect cells against oxidative damage. Dysregulation of these pathways has been implicated in multiple diseases, such as degenerative disorders, ischemic organ injuries, and cancer. In cancer, ferroptosis offers new insights into tumor suppression mechanisms.

Cancer remains a leading cause of global mortality, imposing a significant burden on healthcare systems and patients. Despite advances in treatment modalities, such as targeted therapy and immunotherapy, the development of pharmacological resistance poses a critical challenge, undermining treatment efficacy and leading to disease recurrence and metastasis. This highlights an urgent need for novel therapeutic targets to overcome drug resistance.

Recent research has demonstrated the pivotal role of ferroptosis in drug-resistant cancers, positioning ferroptosis-related signaling pathways as promising therapeutic targets^[3]^. For instance, targeting key regulators, such as GPX4 or Sx C-, has shown potential in sensitizing cancer cells to conventional therapies^[4,5]^. This review aims to summarize the role of ferroptosis in cancer drug resistance, focusing on its underlying signaling pathways and exploring therapeutic strategies to modulate ferroptosis for improved treatment outcomes.

## AN OVERVIEW OF FERROPTOSIS

Ferroptosis, identified by Dixon *et al.* in 2012, was initially observed during the screening of rat sarcoma (RAS)-specific lethal small molecules that triggered non-apoptotic cell death. Erastin was found to trigger ferroptosis via blockage of the Sx C-, an amino acid antiporter responsible for exchanging extracellular cystine and intracellular glutamate at a 1:1 ratio^[1,6]^. This exchange is critical for maintaining intracellular redox homeostasis by supporting glutathione (GSH) synthesis. Subsequent studies identified GPX4 as a pivotal enzyme that prevents lipid peroxidation, with its inhibition promoting ferroptosis, emphasizing its central role in regulating this cell death process^[7]^. The significance of iron and lipid metabolism in rendering cells susceptible to ferroptosis was also highlighted. Other crucial mediators, such as acyl-coenzyme A synthetase long-chain family member 4 (ACSL4), ferroptosis suppressor protein 1 (FSP1), dihydroorotate dehydrogenase and vitamin K, have been identified, further linking ferroptosis to pathological conditions like cancer^[8-12]^. Given the genetic mutations, altered metabolic states, and dysregulated ferroptosis defense systems in cancer, tumor cells exhibit a heightened vulnerability to ferroptosis, presenting new therapeutic opportunities^[3]^.

## MECHANISM OF CANCER DRUG RESISTANCE

Cancer drug resistance refers to the capacity of cancer cells to withstand the cytotoxic effects of anticancer agents, significantly diminishing treatment efficacy and contributing to cancer recurrence and progression. Resistance can manifest as intrinsic resistance, where cancer cells are inherently unresponsive to treatment, or acquired resistance, which develops due to selective pressures during therapy. The mechanisms underlying cancer drug resistance are complex and multifactorial, including alterations in drug targets, increased drug efflux through transporters such as ATP-binding cassette family, drug inactivation, changes in the cell cycle, and evasion of apoptotic pathways through downregulation of pro-apoptotic proteins like BCL2-associated X^[13]^. Beyond these cellular mechanisms, the tumor microenvironment plays a critical role in fostering resistance. Hypoxia, stromal cell interactions, and extracellular matrix components create protective niches that shield cancer cells from therapeutic agents^[14]^. Understanding these diverse resistance mechanisms is essential for developing strategies to overcome drug resistance, such as combination therapies targeting multiple pathways or exploiting vulnerabilities in resistant cancer cells.

## FERROPTOSIS-RELATED SIGNALING PATHWAYS IN CANCER DRUG RESISTANCE

### Oxidative damage-related signaling pathways

#### Canonical GPX4-regulated signaling pathway

**Figure 1:**
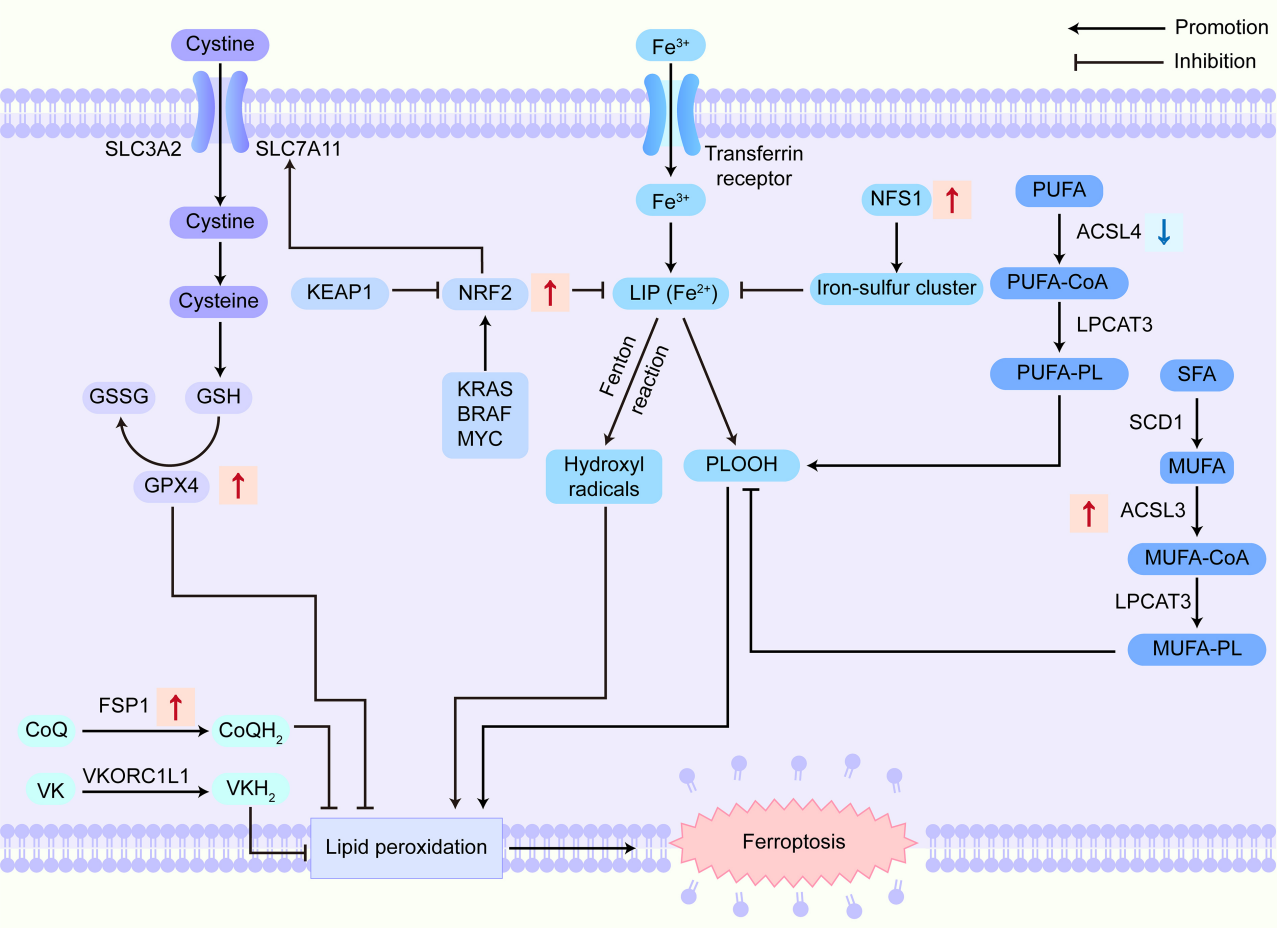
Ferroptosis-related signaling pathways in cancer drug resistance. The signaling pathways that promote ferroptosis mainly include those related to the oxidant system, iron toxicity, and the production and peroxidation of PUFA-PLs, while the signaling pathways that inhibit ferroptosis primarily involve GPX-dependent and FSP1-dependent antioxidant signaling pathways, as well as those related to MUFA-PL synthesis. In cancer drug resistance, pro-ferroptosis key factors such as ACSL4 are downregulated, while anti-ferroptosis factors like GPX4, NRF2, FSP1, NFS1, and ACSL3 are upregulated. SLC3A2: Solute carrier family 3 member 2; SLC7A11: solute carrier family 7 member 11; GSH: glutathione; GSSG: oxidized glutathione; GPX4: glutathione peroxidase 4; CoQ: coenzyme Q; CoQH_2_: reduced coenzyme Q; FSP1: ferroptosis suppressor protein 1; VK: vitamin K; VKH_2_: reduced vitamin K; VKORC1L1: vitamin K epoxide reductase complex subunit 1-like 1; NRF2: nuclear factor E2-related factor 2; KEAP1: Kelch-like ECH-associated protein 1; KRAS: Kirsten rat sarcoma viral oncogene homolog; BRAF: B-Raf proto-oncogene; MYC: myelocytomatosis oncogene; LIP: labile iron pool; PLOOH: phospholipid hydroperoxides; NFS1: cysteine desulfurase; PUFA: polyunsaturated fatty acid; CoA: coenzyme A; ACSL4: acyl-CoA synthetase long-chain family member 4; PL: phospholipid; LPCAT3: lysophosphatidylcholine acyltransferase 3, incorporating fatty acids especially PUFAs into PLs; SFA: saturated fatty acid; MUFA: monounsaturated fatty acid; SCD1: stearoyl-CoA desaturase 1; ACSL3: acyl-CoA synthetase long-chain family member 3, activating MUFAs.

GPX4, a key member of the glutathione peroxidase family, plays a critical role in the modulation of ferroptosis by converting lipid hydroperoxides into inert lipid alcohols, thus preventing their accumulation and protecting cellular membranes. Its anti-ferroptotic function depends on GSH as a cofactor, with its activity regulated by the Sx C-^[7]^. This system, composed of solute carrier family 3 member 2 (SLC3A2) and solute carrier family 7 member 11 (SLC7A11), facilitates cystine uptake, which is crucial for GSH synthesis [Figure 1].

In cancer, GPX4 exhibits a complex role. It often helps tumor cells resist ferroptosis, allowing them to survive oxidative stress and evade cell death, thereby contributing to ferroptosis resistance and drug resistance. Many cancers elevate GSH levels and activate GPX4 to counteract oxidative damage caused by therapies^[15]^. GPX4 inhibition effectively sensitizes resistant cancer cells to ferroptosis, particularly in malignancies such as clear-cell carcinomas, where oxidative stress is a critical vulnerability^[16]^. Preclinical evaluations of GPX4 inhibitors, including RAS-selective lethal 3 (RSL3) and ML162, demonstrate promising potential in selectively inducing ferroptosis in GPX4-dependent tumors^[17]^. Therefore, targeting GPX4 has emerged as a viable strategy to enhance ferroptosis-mediated tumor cell death in resistant tumor cells.

#### Antioxidant system

The transcription factor NRF2 modulates detoxification, antioxidant defense, and drug metabolism by upregulating genes with antioxidant response elements. Kelch-like ECH-associated protein 1 (KEAP1) negatively regulates NRF2 activity by binding to its Neh2 domain, which contains two motifs, ETGE and DLG, with distinct binding affinities^[18]^. KEAP1 mediates NRF2 degradation via ubiquitination, maintaining its low cellular levels. Under stress, NRF2 is rapidly activated to maintain homeostasis and counteract external insults. However, in cancer cells, mutations in KEAP1 or NRF2 hyperactivate NRF2, protecting them from oxidative stress and therapy-induced damage^[19]^. Therefore, targeting NRF2, through strategies like NRF2-siRNA or KEAP1 overexpression, has gained attention as a promising approach to sensitize cancer cells to treatment.

#### Iron metabolism

Iron homeostasis is governed by two key iron-regulating proteins (IRPs), IRP1 and IRP2, which tightly coordinate transferrin receptor and ferritin expression to regulate labile iron levels^[20]^. Ferroptosis is marked by elevated ferrous iron (Fe^2+^) levels in the labile iron pool (LIP). The enhanced LIP generates hydroxyl radicals via the Fenton reaction, where Fe^2+^ reacts with hydrogen peroxide (H_2_O_2_) to form highly reactive hydroxyl radicals (•OH), and contributes to phospholipid peroxidation by forming phospholipid hydroperoxides (PLOOH), which can be prevented by sequestering LIP into the iron-sulfur cluster. The iron-sulfur cluster is a protein cofactor sensitive to oxidative damage and its biosynthesis depends on cysteine desulfurase (NFS1), an enzyme crucial for iron-sulfur cluster formation^[21]^. Therefore, inhibition of NFS1 impairs iron-sulfur cluster biosynthesis, elevating LIP levels and enhancing ferroptosis. High iron levels in rapidly proliferating cancer cells make iron metabolism manipulation a promising approach to overcoming resistance.

Autophagy facilitates iron metabolism by degrading ferroptosis repressors like ferritin through ferritinophagy, increasing iron availability, lipid peroxidation and ferroptosis^[22]^. This mechanism helps overcome sorafenib resistance in hepatocellular carcinoma^[23]^. However, autophagy’s dual role complicates its targeting: while excessive autophagy promotes ferroptosis, it can also support cancer cell survival, particularly in drug-resistant contexts. For instance, autophagy inhibition sensitizes glioblastoma stem-like cells to temozolomide by inducing ferroptosis, offering a strategy to address glioblastoma resistance^[24]^.

Iron chelation deprives tumor cells of essential iron, with common chelators like deferoxamine, deferiprone, and deferasirox showing therapeutic potential^[25]^. Curcumin, a polyphenolic chelator, ameliorates oxidative stress, reduces ferroptosis, and mitigates the tumor-promoting effect of iron overload in normal cells^[26,27]^. Interestingly, in cancer cells, curcumin not only regulates iron levels but also disrupts antioxidant pathways such as GPX4/GSH, thereby promoting ferroptosis, suggesting a tissue- and dose-dependent action^[26,28]^. Despite its promise, curcumin’s mechanisms remain poorly understood, warranting further investigation into its role in drug resistance.

#### Lipid metabolism

Lipid peroxidation, central to ferroptosis, involves both enzymatic and nonenzymatic pathways. In nonenzymatic lipid peroxidation, ACSL4 conjugates polyunsaturated fatty acid (PUFA) to coenzyme A (CoA) to form PUFA-CoA, which lysophosphatidylcholine acyltransferases re-esterify into phospholipids [Figure 1]^[29]^. Elevated ACSL4 correlates with increased ferroptosis sensitivity^[10]^ and enhanced invasiveness in cancers such as colorectal cancer^[30]^. By contrast, established as ferroptosis suppressors, monounsaturated fatty acids (MUFAs) are much less oxidizable, with stearoyl-CoA desaturase-1 (SCD1) being the synthesis rate-limiting enzyme^[31]^. A competitive dynamic exists between PUFA and MUFA metabolism. Conditions or treatments favoring the uptake, biosynthesis, or incorporation into membrane phospholipids of MUFAs rather than PUFAs can decrease sensitivity to ferroptosis^[32]^. Enzymatic lipid peroxidation involves key enzymes such as lipoxygenases, cytochrome P450 oxidoreductase, and cytochrome b5 oxidoreductase. Lipoxygenases generate hydroperoxides within the cellular pool, sensitizing cells to ferroptosis^[33]^. Cytochrome P450 oxidoreductase and cytochrome b5 oxidoreductase promote PUFA peroxidation and ferroptosis induction via H_2_O_2_ generation^[34]^. These above processes lead to the production of PLOOH and the accumulation of 4-hydroxynonenal or malondialdehyde, which are oxidative damage-induced lipid peroxidation biomarkers. These alterations destabilize cellular membranes and drive ferroptosis.

Altered lipid metabolism in resistant cancer cells reduces ferroptosis sensitivity, complicating therapies that rely on its induction^[35]^.

### Crosstalk between ferroptosis and cancer-related signaling pathways

The crosstalk between ferroptosis and key cancer pathways significantly influences therapy resistance, underscoring the need for strategies to exploit ferroptosis in cancer treatment.

#### RAS

RAS is the first oncogene implicated in ferroptosis, with erastin and RSL3 initially identified through a RAS synthetic lethal screen. These ferroptosis inducers specifically target engineered RAS mutant tumor cells via the RAS-BRAF (B-rapidly accelerated fibrosarcoma)-MAPK (mitogen activated protein kinase)/MEK (mitogen-activated protein kinase kinase)-MAPK/ERK (extracellular signal-regulated kinase) pathway and voltage-dependent anion channel (VDAC), inducing oxidative stress and mitochondrial dysfunction^[1,36,37]^. Blocking RAS or its downstream RAF/MEK/MAPK signaling cascade reverses the cytotoxicity caused by erastin or RSL3, likely due to mutant RAS signaling boosting cellular basal iron by regulating the genes involved in iron metabolism^[36,37]^. These findings support ferroptosis induction as a potential strategy for overcoming RAS-driven cancer resistance. However, certain RAS mutations, such as mutant Kirsten rat sarcoma virus (KRAS) in lung cancers, diminish ferroptosis sensitivity, highlighting the role of tumor context and specific mutations in determining ferroptosis susceptibility^[38,39]^.

#### TP53

In addition to RAS, p53 exerts dual effects on ferroptosis. A conventional view is that p53 functions as a tumor suppressor via transcriptional inhibition of SLC7A11 and vitamin K epoxide reductase complex subunit 1-like 1, sensitizing cells to ferroptosis^[40-42]^. Mutations or polymorphisms in TP53, such as the p53^4KR^ (K98R + 3KR) variant, can impair its ability to induce ferroptosis, leading to the loss of tumor suppressor function^[43,44]^. Reversing p53 degradation could overcome ferroptosis resistance^[45]^. There is also evidence suggesting that p53 suppresses ferroptosis in specific cancer types. For example, in colorectal cancer, p53 non-transcriptionally inhibits dipeptidyl peptidase 4 activity to prevent Nicotinamide adenine dinucleotide phosphate (NADPH) oxidase-mediated lipid peroxidation and erastin-induced ferroptosis, whereas TP53 deficiency enhances this process^[46,47]^. Furthermore, p53 mitigates ferroptosis by upregulating CDKN1A expression in fibrosarcoma cells^[48]^. These findings position p53 as a key regulator and therapeutic target for ferroptosis resistance.

#### NFE2L2

Similarly, the NFE2L2 pathway is another crucial player in regulating ferroptosis. The NFE2L2 pathway, often upregulated in cancers, regulates antioxidant response and ferroptosis resistance. Under normal conditions, KEAP1 represses NRF2, forming part of an E3 ubiquitin ligase complex that targets NRF2 for ubiquitination and proteasome-dependent degradation. KEAP1, frequently mutated in cancer, functions as a tumor suppressor^[49,50]^. Furthermore, the expression of endogenous oncogenic alleles such as KRAS, BRAF, and Myelocytomatosis oncogene (MYC) increases NRF2 transcription, thereby elevating the basal NRF2 antioxidant program^[51]^. NRF2 suppresses early-stage tumor initiation^[51]^. However, elevated constitutive levels of NRF2 in cancer cells, following oncogenic driver mutations, possibly contribute to drug resistance^[52,53]^. NRF2’s regulatory role in ferroptosis is impacted by Fe^2+^ within cancer cells. Specifically, in breast cancer cells with high Fe^2+^ concentrations, protein arginine methyltransferase 5 targets the NRF2/heme oxygenase 1 (HMOX1) axis and slows down the ferrous import^[54]^. Therefore, further study is required to explore NRF2’s role in ferroptosis and drug resistance under distinct conditions.

### Regulation of ferroptosis-related signaling pathways in cancer drug resistance

#### GPX4 and FSP1 signaling

As mentioned earlier, GPX4 is a GSH-dependent ferroptosis inhibitor that reduces lipid peroxides, whereas FSP1 functions independently of GSH, which acts as an oxidoreduction enzyme reducing coenzyme Q (CoQ) to ubiquinol (CoQH2) at the cell membrane. CoQH2 serves as a lipophilic antioxidant, trapping free radicals and inhibiting lipid peroxides^[8]^. Both GPX4 and FSP1 are key contributors to tumor resistance, especially GPX4.

In nasopharyngeal carcinoma, Epstein-Barr virus infection triggers the p62-KEAP1-NRF2 axis, upregulating SLC7A11 and GPX4, which decreases sensitivity to ferroptosis. GPX4 modulates TAK1 kinase activity and activates downstream pathways including MAPK-JNK (Jun N-terminal kinase) and nuclear factor kappa B (NFκB) by interacting with the TAK1-TAB1/TAB3 complex, thus reducing sensitivity to cisplatin and paclitaxel^[55]^. In glioblastoma, Selenoprotein P maintains GPX4 levels, contributing to chemoresistance^[56]^. In non-small cell lung cancer, NRF2 upregulates GPX4 and superoxide dismutase 2, conferring resistance to epidermal growth factor receptor tyrosine kinase inhibitors^[57]^, which can be overcome by inhibiting GPX4 to induce ferroptosis^[58]^. Lactate-induced mitochondrial ROS production activates the p38-SGK1 pathway, weakening neural precursor cell expressed, developmentally down-regulated 4-like E3 ubiquitin protein ligase (NEDD4L)’s interaction with GPX4, contributing to etoposide resistance^[59]^. In breast cancer, RelB-mediated GPX4 upregulations drive tamoxifen resistance^[15]^. In gastric cancer, the Wnt/β-catenin signaling pathway enhances GPX4 expression through the β-catenin/TCF4 transcription complex, thereby suppressing ferroptosis. Conversely, TCF4 deficiency promotes cisplatin-induced ferroptosis^[60]^. Colorectal cancer exhibits oxaliplatin resistance mediated by Fusobacterium nucleatum via the E-cadherin/β-catenin/TCF4/GPX4 axis, and the KIF20A/NUAK1/PP1β/GPX4 pathway may further support this resistance^[61,62]^. Hepatocellular carcinoma demonstrates sorafenib resistance through PLAG1-GPX4 interactions, which inhibits sorafenib-triggered ferroptosis via the PVT1/miR-195-5p pathway^[63]^, with additional contributions from the NeuroD1-GPX4 pathway^[64]^.

Moreover, FSP1 confers ferroptosis resistance in KEAP1-mutant non-small cell lung cancer through both NRF2-dependent and -independent mechanisms^[65]^. Patients with head and neck squamous cell carcinoma who experience recurrence post-cisplatin treatment show high FSP1 levels, with cisplatin also inducing the FSP1/ACSL4 axis^[66]^. In pancreatic cancer, the LINC01133-FUS-FSP1 complex stabilizes FSP1 mRNA, driving sorafenib resistance^[67]^. In radioresistant tumor cells, CoQ upregulation shifts ferroptosis inhibition from GPX4 to FSP1, promoting radiotherapy resistance^[68]^.

#### AMP-activated protein kinase signaling

Besides GPX4 and FSP1, another key regulator of ferroptosis resistance is AMP-activated protein kinase (AMPK), which exhibits dual roles across various cancers. In hepatocellular carcinoma, AMPK activation by ferroptosis inducers like erastin, sorafenib, and sulfasalazine inhibits branched chain amino acid transaminase 2 (BCAT2) transcription, modulating glutamate levels and preventing ferroptosis^[69]^. Conversely, lactate-rich hepatocellular carcinoma cells show resistance to ferroptosis, where monocarboxylate transporter 1 (MCT1)-mediated lactate uptake inactivates AMPK and subsequently upregulates sterol regulatory element binding protein 1 (SREBP1) and SCD1, leading to increased production of anti-ferroptotic MUFAs^[70]^. In colorectal cancer, TP53-induced glycolysis regulatory phosphatase (TIGAR) drives resistance to erastin-induced ferroptosis via the ROS/AMPK/SCD1 pathway^[71]^, while inhibiting protein phosphatase 2 catalytic subunit alpha sensitizes these cells to ferroptosis by suppressing the AMPK/SCD1 axis^[72]^. Additionally, in melanoma, arachidonate 5-lipoxygenase induces autophagy-dependent ferroptosis via the AMPK/mammalian target of rapamycin (mTOR) pathway and downregulated GPX4^[73]^, whereas calcium/calmodulin-dependent protein kinase kinase 2 protects cells against ferroptosis through the AMPK-NRF2 pathway^[74]^. These findings underscore AMPK’s complex interplay with ferroptosis, offering potential therapeutic targets.

#### Hypoxia signaling

Hypoxia also drives ferroptosis resistance and chemoresistance by regulating hypoxia-inducible transcription factors (HIFs) and downstream pathways. HIFs modulate gene expression linked to glucose uptake and utilization, angiogenesis, and erythropoiesis^[75]^. Comprising a constitutively expressed subunit and an oxygen-labile subunit, HIFs are highly expressed in cancer cells, potentially due to their rapid proliferation and increased oxygen consumption^[76]^. HIFs play dual roles in ferroptosis. In fibrosarcoma cells, HIF1A promotes lipid storage and reduces fatty acid oxidation by increasing fatty acid binding proteins 3 and 7, which prevents ferroptosis^[77]^. Conversely, HIF-2α induces an iron-death-sensitive state in colorectal cancer cells via upregulation of lipid- and iron-regulated genes and enhanced ROS production, increasing cell death^[78]^.

Beyond HIFs, hypoxia impacts ferroptosis and resistance through other mechanisms. In esophageal squamous cell carcinoma, hypoxia inhibits ferritinophagy-mediated ferroptosis through the USP2 (ubiquitin specific peptidase 2)-NCOA4 (nuclear receptor coactivator 4) axis^[79]^. In gastric cancer, hypoxia-induced lncRNA-CBSLR protects cells against ferroptosis, resulting in chemoresistance^[80]^. In hepatocellular carcinoma, the hypoxia-responsive PPARGC1A (peroxisome proliferator activated receptor gamma coactivator 1 alpha)/BAMBI (bone morphogenetic protein and activin membrane bound inhibitor)/ACSL5 (acyl-CoA synthetase long chain family member 5) pathway confers lenvatinib resistance^[81]^. In pancreatic ductal adenocarcinoma, hypoxia induces DNA methyltransferase 3 beta overexpression, leading to hypermethylation of the miR-485-3p promoter, alleviating SLC7A11 inhibition and promoting chemoresistance^[82]^. Conversely, in nasopharyngeal carcinoma, hypoxia-induced BRCA1-associated deubiquitinase 1 stabilizes H2A, sensitizing cells to erastin-induced ferroptosis and improving chemoresistance^[83]^.

#### PI3K-AKT-mTOR signaling

The PI3K-AKT-mTOR pathway centrally regulates cell growth, survival, and metabolism, and its hyperactivation is a key driver of resistance to chemotherapy and ferroptosis. Specifically, high activity of the PI3K-AKT-mTOR axis keeps cancer cells from oxidative stress and ferroptosis via SREBP1/SCD1-mediated lipogenesis. Preclinical models suggest that inhibiting mTORC1 while inducing ferroptosis could be a viable strategy^[84]^. G protein-coupled estrogen receptor 1 (GPER1), which reduces H_2_O_2_ cytotoxicity and decreases sensitivity to the ferroptosis inducer RSL3, impairs lipid peroxidation in non-small cell lung cancer. GPER1, along with its agonist G1, upregulates SCD1 expression and activates the PI3K/AKT/mTOR signaling. GPER1 knockdown enhances cisplatin efficacy, highlighting its potential as a therapeutic target to overcome resistance^[85]^.

#### Interactions between ferroptosis-related signaling pathways in cancer drug resistance

Notably, interactions among ferroptosis-related pathways, especially GPX4, NRF2, and iron metabolism regulators, are pivotal in cancer drug resistance. NRF2 upregulates SLC7A11, enhances GSH synthesis, and supports GPX4 activity, driving radio- and chemoresistance in cancers such as esophageal squamous cell carcinoma, clear cell renal cell carcinoma, lung adenocarcinoma, and nasopharyngeal carcinoma^[55,86-88]^. Targeting the NRF2/GPX4 axis sensitizes cancer cells to ferroptosis and improves therapy efficacy, as seen with sorafenib in hepatocellular carcinoma and oxaliplatin in colorectal cancer^[62,89]^. NRF2 also regulates LIP through proteins like HERC2 and VAMP8. NRF2-deficient cells exhibit decreased HERC2 (homologous to the E6-AP carboxyl terminus and regulator of chromosome condensation 1-like domain containing E3 ubiquitin protein ligase 2) and VAMP8 (vesicle associated membrane protein 8), leading to apoferritin accumulation and elevated LIP, which sensitizes cells to ferroptosis. Thus, targeting NRF2 offers a therapeutic approach to overcoming drug resistance^[90]^.

## TARGETING FERROPTOSIS-RELATED SIGNALING PATHWAYS TO OVERCOME CANCER DRUG RESISTANCE

### Chemoresistance

**Figure 2:**
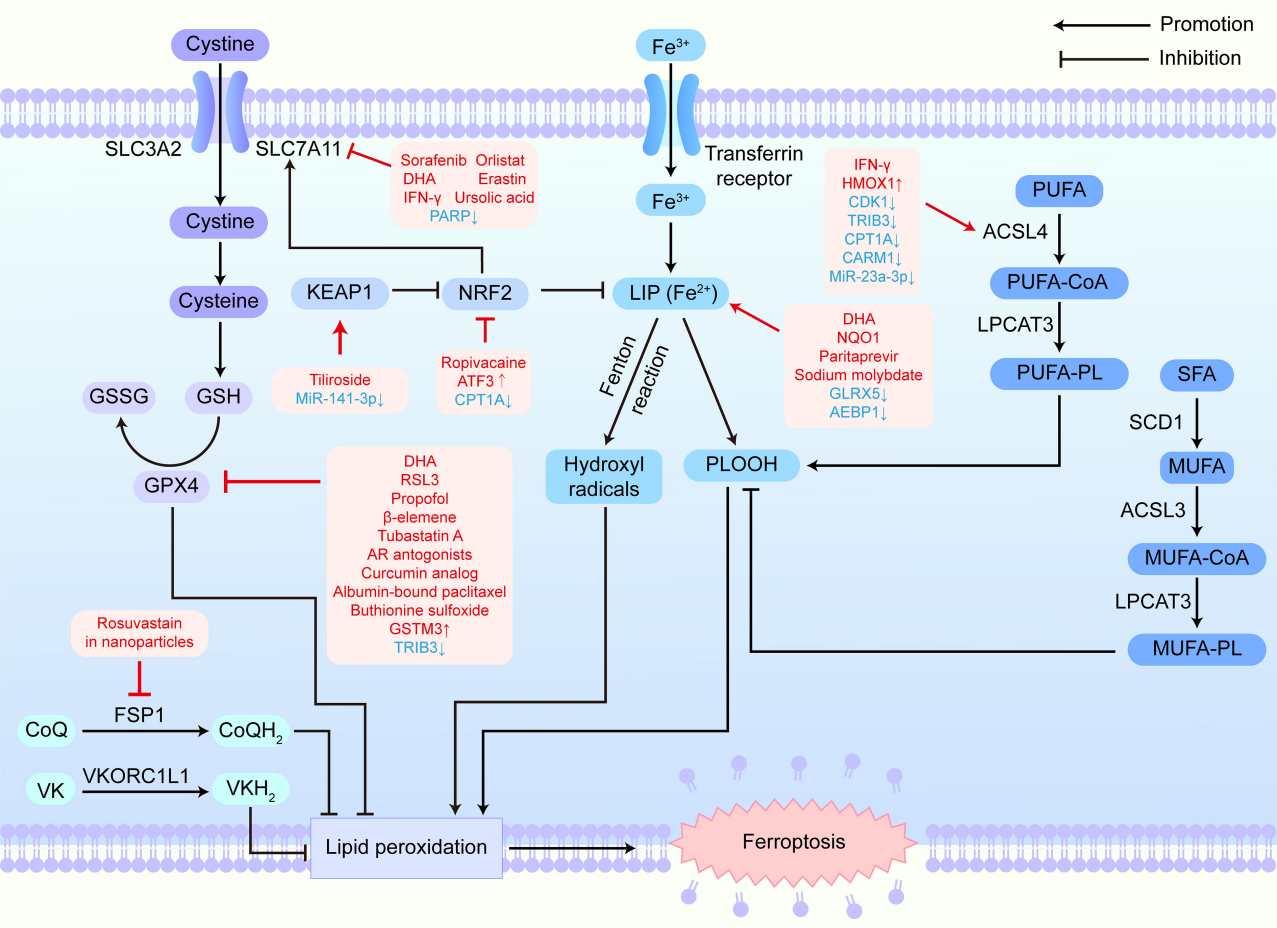
Targeting ferroptosis-related signaling pathways to overcome cancer drug resistance. Promoting pro-ferroptosis signaling pathways or inhibiting anti-ferroptosis signaling pathways by targeting key factors such as GPX4, NRF2, SLC7A11, and ACSL4 predisposes cancer cells to ferroptosis and reduces drug resistance. SLC3A2: Solute carrier family 3 member 2; SLC7A11: solute carrier family 7 member 11; GSH: glutathione; GSSG: oxidized glutathione; GPX4: glutathione peroxidase 4; CoQ: coenzyme Q; CoQH_2_: reduced coenzyme Q; FSP1: ferroptosis suppressor protein 1; VK: vitamin K; VKH_2_: reduced vitamin K; VKORC1L1: vitamin K epoxide reductase complex subunit 1-like 1; NRF2: nuclear factor E2-related factor 2; KEAP1: Kelch-like ECH-associated protein 1; LIP: labile iron pool; PLOOH: phospholipid hydroperoxides; PUFA: polyunsaturated fatty acid; CoA: coenzyme A; ACSL4: acyl-CoA synthetase long-chain family member 4; PL: phospholipid; LPCAT3: lysophosphatidylcholine acyltransferase 3, incorporating fatty acids especially PUFAs into PLs; SFA: saturated fatty acid; MUFA: monounsaturated fatty acid; SCD1: stearoyl-CoA desaturase 1; ACSL3: acyl-CoA synthetase long-chain family member 3, activating MUFAs; IFN: interferon; PARP: poly (ADP-ribose) polymerase; DHA: dihydroartemisinin; AR: androgen receptor; GSTM3: glutathione S-transferase mu 3; TRIB3: tribbles pseudokinase 3; ATF3: activating transcription factor 3; CPT1A: carnitine palmitoyl transferase 1A; HMOX1: heme oxygenase 1; CDK1: cyclin dependent kinase 1; CARM1: coactivator-associated arginine methyltransferase 1; NQO1: NAD(P)H:quinone oxidoreductase 1; GLRX5: glutaredoxin 5; AEBP1: adipocyte enhancer binding protein 1.

Numerous chemotherapeutic agents exert antitumor effects by triggering ferroptosis. Abnormal ferroptosis contributes to chemoresistance, while strategies to modulate ferroptosis have shown promise in mitigating this resistance. Key signaling pathways involved in reversing chemotherapy resistance include the GPX4-regulated pathway, oxidant and antioxidant system, iron metabolism, and lipid metabolism, all of which offer novel approaches to enhance chemotherapy efficacy [Figure 2].

#### Targeting canonical GPX4-regulated pathway

GPX4 activation suppresses ferroptosis and confers chemoresistance. For example, RelB-mediated GPX4 upregulation facilitates tamoxifen resistance in breast cancer^[15]^, while GPX4 inactivation induces ferroptosis and augments chemotherapy sensitivity. For instance, curcumin analogs promote androgen receptor (AR) ubiquitination, disrupting GPX4-mediated redox homeostasis and inhibiting temozolomide-resistant glioblastoma growth^[91]^. Similarly, albumin-bound paclitaxel sensitizes glioblastoma cells to temozolomide by downregulating GPX4 and impairing DNA damage repair^[4]^. A GPX4 inhibitor has been shown to boost platinum agent efficacy in lung cancer brain metastases^[92]^. Dihydroartemisinin (DHA) enhances GPX4 inhibition-induced ferroptosis by increasing free iron levels, sensitizing ferroptosis-resistant cancer cells^[93]^. Furthermore, propofol mitigates cisplatin resistance in non-small cell lung cancer by downregulating GPX4 and inducing ferroptosis via the miR-744-5p/miR-615-3p pathway^[94]^.

Additionally, nanotechnology has advanced ferroptosis-based approaches. Specifically, an iron-based ferroptosis-inducing platform co-loaded with buthionine sulfoximine and oxaliplatin alleviates resistance to chemotherapy and enhances oxaliplatin efficacy by blocking GSH biosynthesis and inactivating GPX4^[95]^. Magnetic composite nanoparticles loaded with doxorubicin and DHA synergize to suppress triple-negative breast cancer via the PI3K/AKT/mTOR/GPX4 axis^[96]^. An activatable nanomedicine combats hypoxia-induced chemoresistance in solid tumors by triggering both ferroptosis and apoptosis^[97]^.

#### Targeting antioxidant system

Cancer cells often develop resistance to chemotherapy and ferroptosis by stabilizing NRF2 through KEAP1 inactivation or genetic alterations in the NRF2 pathway, with NRF2 overexpression associated with poor outcomes in primary malignant brain tumors^[98,99]^. Pharmacological inhibition of this pathway restores ferroptosis sensitivity, as seen in cisplatin-resistant head and neck cancer cells treated with artesunate^[100]^. Ropivacaine reduces cisplatin resistance in colorectal cancer by blocking the SIRT1/NRF2 pathway^[101]^. In addition, elevated activating transcription factor 3 enhances the cisplatin sensitivity of gastric cancer through NRF2/KEAP1/SLC7A11 inhibition^[102]^. In breast cancer, miR-141-3p inhibitors ameliorate paclitaxel and RSL3 resistance by elevating KEAP1 expression levels^[103]^.

#### Targeting iron metabolism

Low iron levels restrict ferroptosis and result in chemoresistance, while elevated LIPs enhance ferroptosis susceptibility. For example, lipocalin 2 not only decreases intracellular iron levels but also upregulates GPX4 and SLC7A11^[104]^. Strategies to combat drug resistance, such as inducing NAD(P)H:quinone oxidoreductase 1-mediated ferroptosis, elevate ROS production, increase LIP, and induce lipid peroxidation^[105]^. NRF2 deletion also increases LIP, boosting ferroptosis sensitivity^[90]^. The combination of DHA and cisplatin synergistically modulates iron metabolism and triggers ferroptosis in pancreatic ductal adenocarcinoma^[106]^. Inhibiting the KLF5 (Kruppel-like factor 5)/LIF (leukemia inhibitory factor)/MTF1 (metal regulatory transcription factor 1)/FPN1 (ferroportin-1) axis induces iron overload, sensitizing colorectal cancer to oxaliplatin^[107]^. Blocking glutaredoxin 5 or adipocyte enhancer binding protein 1 increases free iron levels, reversing cisplatin resistance^[108,109]^. In human epidermal growth factor receptor 2 (HER2)-low breast cancer, paritaprevir increases ROS and LIP by preventing VDAC3-derived circular RNA from binding heat shock protein family B (small) member 1 protein, overcoming resistance to trastuzumab deruxtecan ^[110]^. Sodium molybdate also stands out as an attractive candidate for ovarian cancer treatment due to its ability to elevate LIP levels^[111]^.

#### Targeting lipid metabolism

ACSL4 influences cellular lipid composition and ferroptosis execution^[10]^. Increased HMOX1 reverses small-cell lung cancer resistance through mic14 regulation, with observed upregulation of ACSL4 and downregulation of GPX4 and SLC7A11 levels^[112]^. Inhibiting ACSL4 methylation mediated by coactivator-associated arginine methyltransferase 1 enhances ferroptosis in colorectal cancer^[113]^, where prohibiting cyclin-dependent kinase 1 also alleviates resistance to oxaliplatin by modulating ACSL4-mediated ferroptosis^[114]^. Tailored lipid and iron presentation can overcome ferroptosis resistance in ACSL4-deficient cancers^[115]^.

SCD1 is another key player in ferroptosis resistance and a promising target to overcome drug resistance^[116]^. Specifically, TIGAR contributes to ferroptosis resistance in colorectal cancer via the ROS/AMPK/SCD1 axis^[71]^, while aspirin enhances RSL3-driven ferroptosis by inhibiting mTOR/SREBP-1 (sterol regulatory element-binding protein-1)/SCD1-regulated lipogenesis in PIK3CA-mutant colorectal cancer^[117]^. Selective deprivation of Zn^2+^, a cofactor of SCD1, sensitizes ovarian cancer to ferroptosis-based treatment^[118]^.

### Targeted therapy resistance

Resistance to targeted therapy can be intrinsic and acquired. Intrinsic resistance often arises from insensitive target variants, mutations in oncogenic pathways, or activation of parallel pathways, while acquired resistance involves alterations at the target site, bypass mechanisms in related pathways, phenotypic changes in tumors, and loss of target dependency^[119]^. Ferroptosis has emerged as a promising strategy to overcome both types of resistance and expand the applicability of targeted therapies.

#### Intrinsic resistance

In KRAS-mutated colorectal cancer, combining cetuximab with β-elemene, a newly established ferroptosis inducer, enhances treatment efficacy by promoting ferroptosis and suppressing epithelial-to-mesenchymal transition^[120]^. Cetuximab also synergizes with RSL3 to overcome KRAS-driven resistance by inhibiting the NRF2/HO-1 axis^[121]^. Similarly, gefitinib resistance is linked to enhanced ferroptosis defense mechanisms^[122,123]^, and inhibiting discoidin domain receptor tyrosine kinase 1 and GPX4 can restore ferroptosis and overcome gefitinib resistance in non-small cell lung cancer and triple-negative breast cancer, respectively^[122,124]^.

Olaparib, a poly (ADP-ribose) polymerase (PARP) inhibitor, is usually employed in the treatment of advanced ovarian cancer patients harboring BRCA1/2 mutations. A recent finding shows that PARP inhibition downregulates SLC7A11, increasing lipid peroxidation and ferroptosis, which works synergistically with ferroptosis inducers in BRCA wild-type ovarian cancer^[125]^. In platinum-resistant ovarian cancer, the combination of arsenic trioxide and olaparib activates the AMPK α pathway and reduces SCD1 expression, ultimately triggering ferroptosis^[126]^.

MBOAT2, a ferroptosis suppressor, is upregulated by AR. Combined therapy of AR antagonists with ferroptosis inducers markedly reduced the growth of AR-positive prostate cancer, even in tumors resistant to hormonal therapy^[127]^. Additionally, TQB3720 activates ferroptosis via the AR/GPX4 pathway in prostate cancer cells^[128]^.

#### Acquired resistance

Sorafenib, a ferroptosis trigger in cancer, often loses efficacy due to factors such as activating transcription factor 2, dipeptidyl peptidase 9, and dual specificity phosphatase 4, which inhibit ferroptosis^[88,129,130]^. Targeting these negative regulators considerably enhances sorafenib sensitivity. Various drugs that drive ferroptosis and synergize with sorafenib have been identified. For instance, combining sorafenib with ursolic acid synergistically induces SLC7A11-dependent ferroptosis in cancer cells^[131]^. Furthermore, tiliroside sensitizes hepatocellular carcinoma to sorafenib by inhibiting tumor necrosis factor receptor-associated factor family member associated nuclear factor-kappa B activator (TANK) binding kinase 1 and inducing ferroptosis^[132]^. MiR-23a-3p, overexpressed among sorafenib non-responders, targets ACSL4 directly and leads to sorafenib resistance, while miR-23a-3p inhibition restores ACSL4 expression and triggers ferroptosis in hepatocellular carcinoma treated with sorafenib^[133]^.

Although sunitinib improves prognosis in renal cell carcinoma, most patients ultimately develop drug resistance, highlighting the need for novel therapeutic targets. Absent in melanoma 2 (AIM2) contributes to sunitinib resistance by regulating ferroptosis through the FOXO3a-ACSL4 pathway, presenting a potential target^[134]^. Moreover, the knockdown of tribbles pseudokinase 3 triggers ferroptosis via the SLC7A11/GPX4 pathway, enhancing sunitinib efficacy in clear cell renal cell carcinoma^[135]^.

Osimertinib resistance remains a critical challenge in non-small cell lung cancer. A novel nanocatalytic sensitizer delivering Vitamin C-Fe (II) effectively overcomes osimertinib resistance and suppresses metastasis, emphasizing its potential in inducing ferroptosis in resistant tumors^[136]^.

### Immunotherapy resistance

Ferroptosis plays a complex role in immunotherapy. Stimulating ferroptosis enhances antitumor immune responses and improves immune checkpoint inhibitor efficacy. For example, targeting carnitine palmitoyl transferase 1A and protein arginine methyltransferase 5 boosts immunotherapy by activating ACSL4 and inhibiting NRF2 [Figure 2]^[54,137]^. Additionally, CD8 T cell-derived interferon-γ suppresses SLC3A2 and SLC7A11, eventually leading to augmented lipid peroxidation and ferroptosis induction^[5]^. Interferon-γ also activates the ACSL4 signaling pathway, promoting fatty acid integration and enhancing ferroptosis^[138]^. Inhibiting the Tyro3 pathway, which is linked to resistance against anti-PD-1 (programmed cell death protein 1)/PD-L1 (programmed death-ligand 1) therapies, further resensitizes tumor cells to immunotherapy by promoting ferroptosis^[139]^. Combining ferroptosis inducers with immunotherapy offers significant potential to enhance antitumor responses and overcome resistance.

Nevertheless, ferroptosis may also trigger immunosuppression and immunotherapy resistance in gliomas due to their unique tumor microenvironment, where ferroptosis inhibition might be beneficial^[140]^.

### Radiotherapy resistance

Ferroptosis is closely tied to radiotherapy resistance due to its interplay with oxidative stress and antioxidant defenses. Ionizing radiation induces oxidative stress, promoting ferroptosis via pathways such as ACSL4 upregulation and ROS generation^[141]^. However, cancer cells counteract this by enhancing antioxidant defenses, notably through GPX4, SLC7A11, and FSP1, which mitigate ferroptosis and sustain cell survival^[141,142]^. NRF2, for instance, drives resistance in esophageal squamous cell carcinoma by activating SLC7A11, reducing oxidative stress, and preventing radiation-induced lipid peroxidation^[143]^. Hypoxia further exacerbates radioresistance by limiting ROS production and activating HIF1-α in cancers such as glioblastoma and rectal cancer^[144,145]^.

Targeting ferroptosis-related signaling pathways could help overcome radiotherapy resistance. Inhibitors of GPX4, such as Tubastatin A and glutathione S-transferase mu 3, enhance radiosensitivity by promoting ferroptosis [Figure 2]^[146,147]^. Notably, FSP1 inhibitors show greater efficacy in radioresistant cells than GPX4 inhibitors, underscoring their therapeutic potential^[68]^. Combinative treatment of ferroptosis inducers and radiotherapy offers a promising strategy to overcome radioresistance.

## CONCLUSION

In summary, ferroptosis has emerged as a promising programmed cell death mechanism for combating cancer drug resistance, enhancing the efficacy of various treatments. To advance ferroptosis-based therapies, a deeper understanding of its signaling pathways is essential for identifying optimal therapeutic targets and minimizing side effects. Identifying cancer types most likely to benefit from such treatments and tailoring therapies to individual tumor profiles will further enhance efficacy and reduce toxicity. Integrating ferroptosis-inducing agents with chemotherapy, targeted therapies, radiotherapy, or immunotherapy could overcome resistance and improve outcomes. Notably, combining ferroptosis with immunotherapy holds promise for boosting antitumor immune responses. For clinical translation, effective biomarkers are needed to monitor ferroptosis, predict therapeutic responses, and manage toxicity. Continued research into ferroptosis mechanisms and their role in resistance will be essential for successful clinical application, paving the way for more targeted and personalized cancer therapies.

## DECLARATIONS

### Copyright

© The Author(s) 2025.
